# Case Report: Resolution of Lichen Planus Pemphigoides as an unexpected outcome of SARS-CoV-2 infection

**DOI:** 10.3389/fimmu.2023.1222459

**Published:** 2023-07-12

**Authors:** Valentina Ruffo di Calabria, Alice Verdelli, Lavinia Quintarelli, Alberto Corrà, Elena Biancamaria Mariotti, Cristina Aimo, Elena Del Bianco, Beatrice Bianchi, Vincenza Maio, Daniela Massi, Marzia Caproni

**Affiliations:** ^1^ Section of Dermatology, Department of Health Sciences, University of Florence, Florence, Italy; ^2^ Rare Skin Diseases Unit, Department of Health Sciences, Azienda USL Toscana Centro (ERN-SKIN), University of Florence, Florence, Italy; ^3^ Section of Anatomic Pathology, Azienda USL Toscana Centro, Florence, Italy; ^4^ Section of Anatomic Pathology, Department of Health Sciences, University of Florence, Florence, Italy

**Keywords:** SARS-CoV-2, COVID-19, autoimmunity, viral infections, Lichen planus pemphigoides, molecular mechanisms

## Abstract

It is well known that viral infections play a relevant role in inducing or protecting from autoimmune diseases, thus representing a major environmental factor in the disruption of the immune system in genetically susceptible individuals. Since the beginning of the Covid-19 pandemic a great number of clinical and epidemiological studies have demonstrated that SARS-CoV-2 infection is no exception to the rule by interfering on many different levels in the normal functioning of our immune system. Even though a growing number of case series and case reports has been cited in the literature linking the infection to the new onset of autoimmune diseases, to date very little has been reported concerning a possible correlation between the virus and the clinical resolution of any kind of autoimmune pathology. Here we describe an interesting case of abrupt and unexpected resolution of Lichen planus pemphigoides mucocutaneous lesions in a fully vaccinated patient after a mildly symptomatic SARS-CoV-2 respiratory infection and we speculate on the possible underlying mechanisms correlating the two events.

## 1 Introduction

Lichen planus (LP) is a common, idiopathic mucocutaneous inflammatory disease usually characterized by chronic evolution. Its clinical hallmark is defined by pruritic, polygonal and violaceous papules histologically associated with typical band-like lymphocytic infiltrate in the upper dermis. According to the distribution and the morphology of the mucocutaneous lesions, many clinical variants of LP can be identified (1). For long time considered as one of them, Lichen planus pemphigoides (LPP) is now recognized as a distinct clinical entity, i.e. an autoimmune sub-epidermal blistering disease which develops in the setting of autoantibodies targeting type XVII collagen (COL17) (2). Both diseases share an intricated pathogenetic background where genetic, environmental and immunogenic factors contribute to the dysregulation of the immune system.

Since the beginning of the Covid-19 pandemic, the literature has been flooded by case reports of patients developing autoimmune and immune-mediated diseases concomitantly or immediately following SARS-CoV-2 infection. New onset of LP following SARS-CoV-2 respiratory infection or vaccinations have also been reported (3), similarly to other previously described association with potential immunological disturbers, such as HCV infection or HBV, HCV and influenza vaccines (4–6). On the other hand, only very few cases have been so far reported of patients affected by immune-mediated disorders who have clinically improved or fully recovered from their condition after a Covid-19 respiratory infection. Here we present the peculiar evolution of a patient with mucocutaneous LP who, subsequently to a mild SARS-CoV-2 infection, first developed LPP lesions confirmed by immunohistopathologic evidence of IgG targeting COL17 antigen, and then very quickly experienced a complete resolution of her overall skin condition.

## 2 Case description

**Figure 1:**
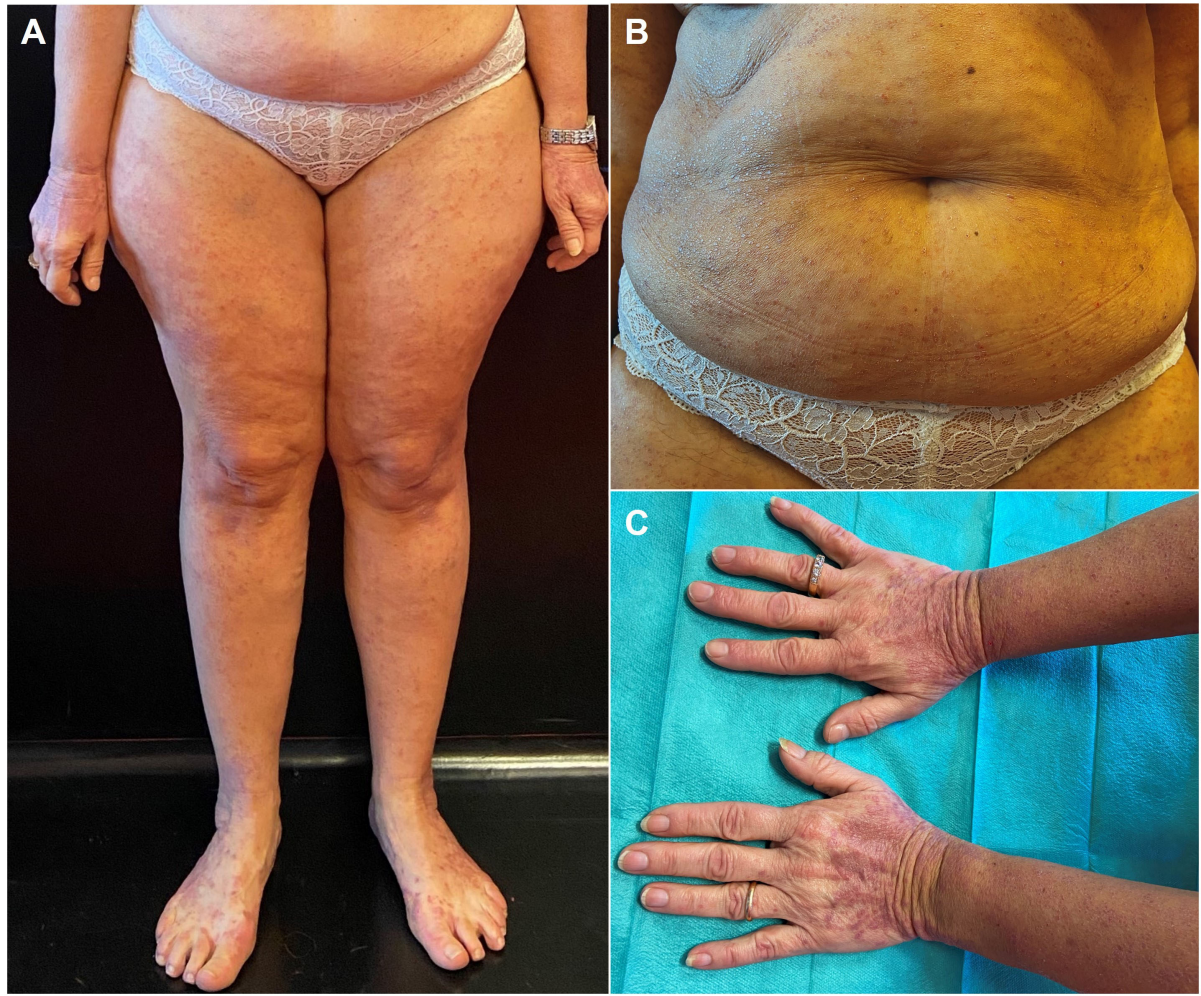
Clinical presentation of our patient at first medical contact. The patient presented with a diffuse cutaneous eruption of violaceous, polygonal, slightly scaling papules on her trunk and limbs **(A, B)** with a marked tendency to coalesce into plaques, especially on the dorsal surface of both her hands and feet **(C)**.

**Figure 2:**
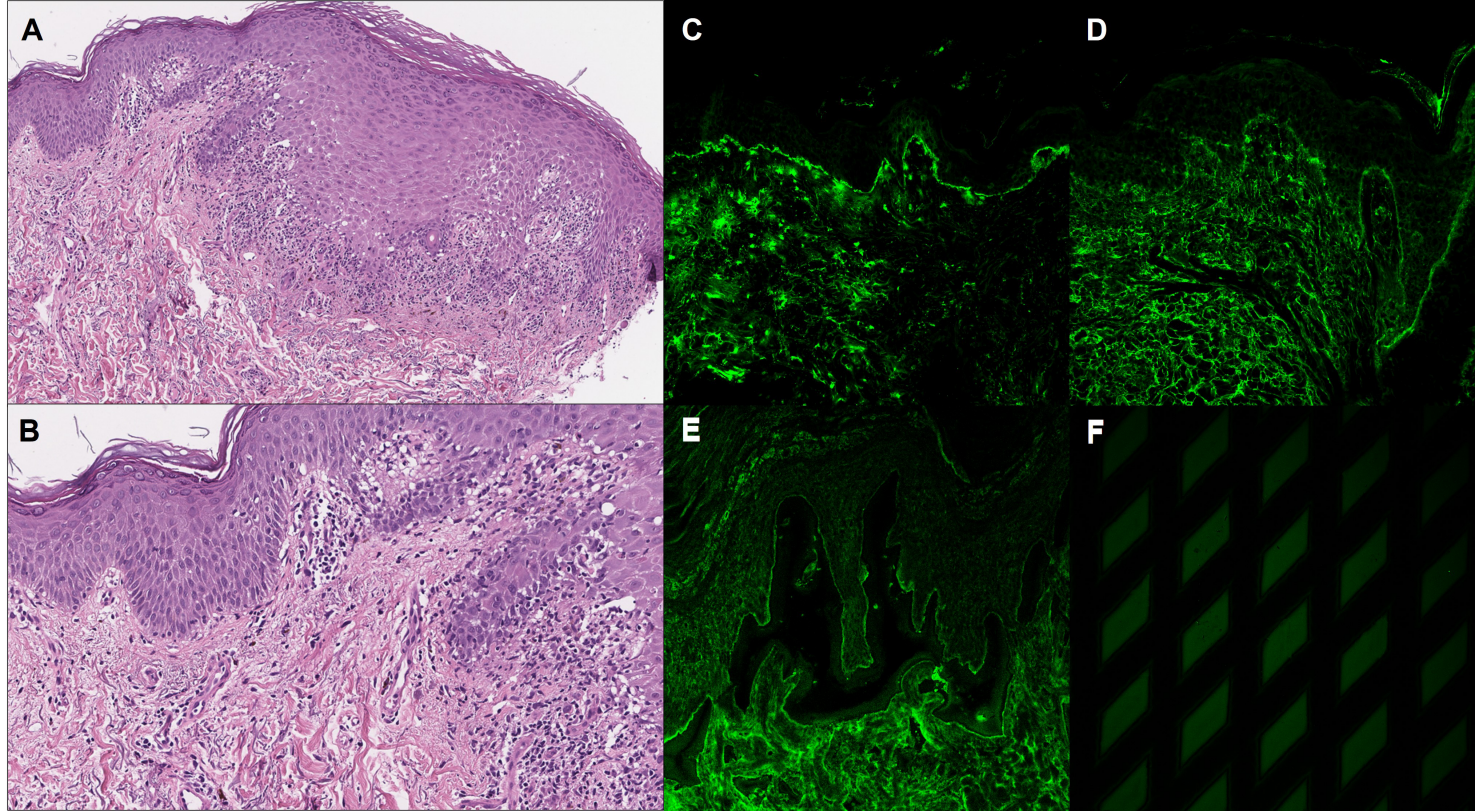
Histopathological and immunopathological findings. **(A, B)** Histopathological examination of a skin lesion on the dorsal surface of the hand showed irregular epidermal hyperplasia with hyperothokeratosis and vacuolar degeneration of basal keratinocytes, while a band-like lymphohistiocytic infiltrate could be found in the upper dermis. Direct immunofluorescence showed linear C3 **(C)** and IgG **(D)** deposits along the dermo-epidermal junction. **(E)** Indirect immunofluorescence on salt-split skin showed IgG linear deposits on the epidermal side of the dermo-epidermal junction. **(F)** Indirect immunofluorescence on Dermatology Mosaic 7 Biochip showed positivity for Ag BP180.

**Figure 3:**
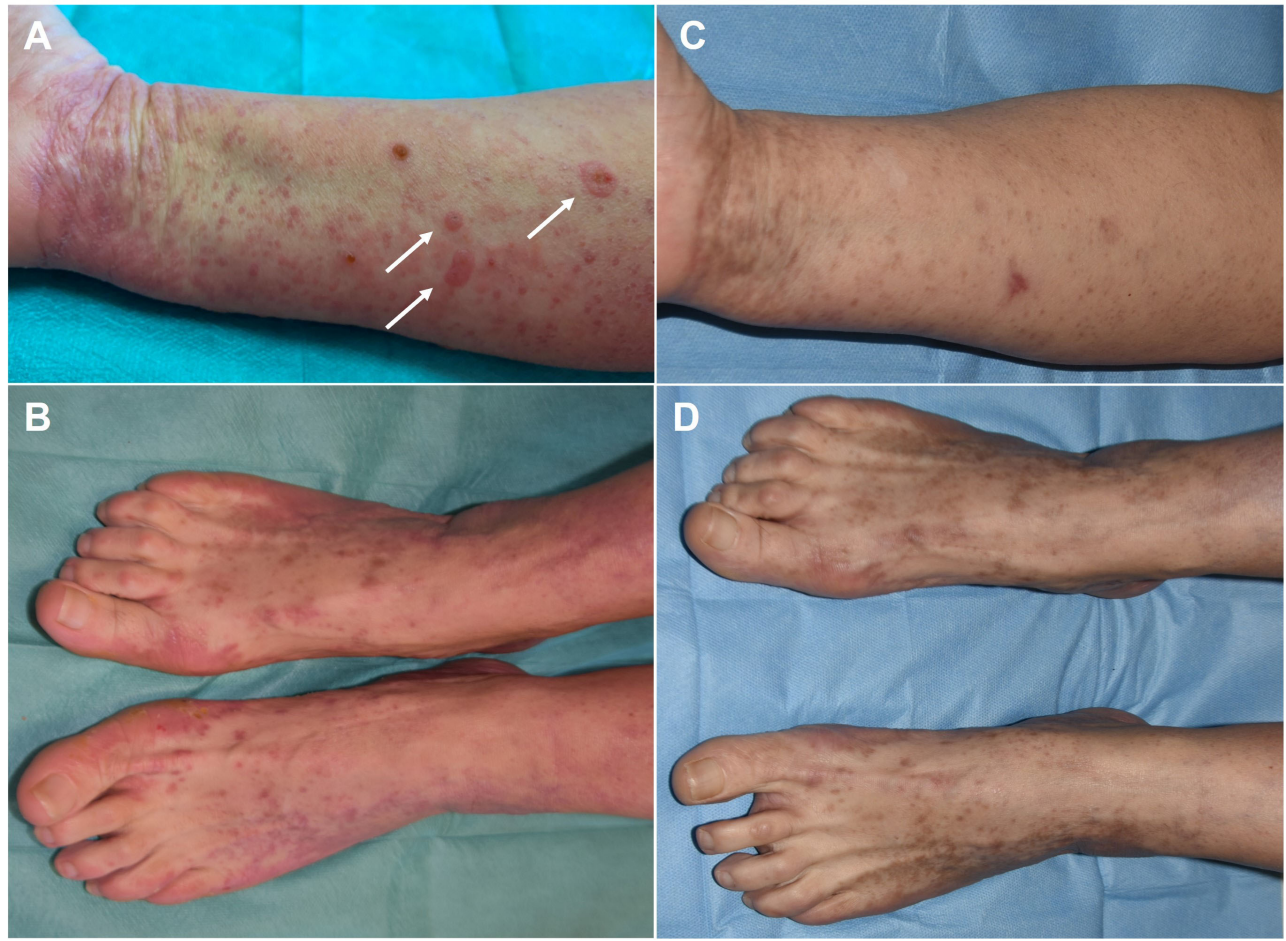
Clinical evolution of our patient. **(A, B)** Clinical pictures immediately following Sars-CoV2 infection when the patients developed tense serous bullae on previously unaffected skin (see arrows). **(C, D)** Clinical pictures of the same areas at 1-week follow-up when only hyperpigmented post-inflammatory marks could be observed.

A 66-year-old woman was referred to our Dermatology clinic in October 2022 complaining of a 2-months history of a diffuse cutaneous eruption that had started with a burning and itching sensation mainly localized to her lower extremities. The patient also recalled of concomitant asymptomatic linear lesions affecting her oral cavity. As soon as she interrupted a 20-days therapy of low dose systemic corticosteroids she experienced a flare in the disease activity that led her to seek for our medical advice. Beside the completion of a 3-doses vaccination cycle for Covid-19 infection in December 2021 and two episodes of asymptomatic and paucisymptomatic Covid-19 infections respectively occurred in September 2020 and April 2022, both her past medical and pharmaceutical anamnesis were negative by the time of the visit. On clinical examination she presented with widespread erythematous papular lesions with violaceous hue disseminated to her trunk and limbs, some of which showed a peculiar polygonal shape and a marked tendency to coalesce into plaques, especially on the dorsal surface of her hands and feet (**Figures 1A–C**). Her mucosae were overall spared except for few painless white lesions on the buccal mucosa that showed a reticular distribution pattern. Based on clinical evaluation and histopathologic evidence of lichenoid tissue reaction (**Figures 2A, B**) a diagnosis of Lichen planus (LP) was made and the patient was started with 25 mg/day of prednisone with a gradual slow tapering every 10 days. Six days after our medical contact the patient suffered from a mild flu-like syndrome, which lasted only 4 days and tested positive to SARS-CoV-2 on molecular nasopharyngeal swab. Concomitant to the onset of the respiratory infection she started developing tense, serous bullae on the inner surface of her right thigh as well as on the volar side of her right forearm, outside the pre-existing lichenoid lesions (**Figures 3A, B**). As soon as the infection resolved further analysis were performed in order to differentiate between the clinical suspicion of Bullous Lichen Planus (BLP) and Lichen Planus Pemphigoides (LPP). Direct immunofluorescence (DIF) on perilesional skin showed IgG and C3 linear deposits at the dermo-epidermal junction and indirect immunofluorescence (IIF) revealed IgG linear deposits on the epidermal side of the dermo-epidermal junction with presence of autoantibodies against the BP180 antigen (**Figures 2C–F**). Despite the evidence collected for a firm diagnosis of LPP, few days after the resolution of the viral infection the patient referred the initial improvement of some of her cutaneous lesions as well as of her long-lasting pruritus. This was confirmed at the following medical contact the next week when most of her skin was surprisingly cleared of bullae and papulae and only diffuse hyperpigmented post-inflammatory marks could be found (**Figures 3C, D**). Even though her cutaneous condition dramatically improved in a very short time, to help the resolution of the last few lesions which persisted on her limbs, the patient was prescribed a weekly dose of 7,5 mg of Methotrexate at the beginning of January 2023. After only three administrations complete clinical remission was obtained. Since then, the patient has fully recovered and hasn’t experienced any flare or relapse of the disease.

Clinical features and treatments during the disease course are summarized in **Table 1**.

**Table 1:** Clinical features and treatment during the disease course.

Days	Clinical features	Treatment
Before visit to our hospital	Burning and itching cutaneous eruption of her legs, asymptomatic linear lesions in her oral cavity.	16 mg/day of methylprednisolone progressively tapered every 5 days (20 days of therapy)
Day 0	Widespread violaceous, polygonal papulae and plaques on her trunk, limbs, dorsal surface of her hands and feet. Few painless white, reticular lesions on her oral mucosa.	25 mg/day of prednisone progressively tapered every 10 days (40 days of therapy)
Day 6	SARS-CoV-2 infection.	
Day 7	Eruption of blisters outside the pre-existing lichenoid plaques.	
Day 13	DIF and IIF conclusive for Lichen planus pemphigoides.	
Day 19	Resolution of her cutaneous lesions, persistence of hyperpigmented post-inflammatory marks.	
Day 52	Persistence of few lesions on her limbs.	7,5 mg/week of methotrexate
Day 70	Clinical remission.	

## 3 Discussion

From the start of the Covid-19 outbreak a growing number of evidence has shown that the virus is able to interfere on many different levels with the normal functioning of our immune system by impairing both its innate and adaptative functions, mainly by eliciting a robust type I IFN response, which is known to be one of the main links between the innate response and the activation of the adaptative immune response (7). Moreover, by being able to induce superantigen activation and autoantibodies production, SARS-CoV-2 acts similarly to other viruses such as cytomegalovirus (CMV), Epstein-Barr virus (EBV) or parvovirus B19, that are known to represent environmental triggers of autoimmunity in genetically predisposed individuals (8). Therefore, it is not surprising that in the last 3 years a flourishing number of case reports has been published concerning patients who developed *de novo* autoimmune diseases, namely type 1 diabetes (T1D), Guillain-Barré syndrome or rheumatoid arthritis (RA), following Covid-19 infection (9–14).

Beside this well-known correlation, it has been demonstrated that viral infections can also play a protective or resolving role in the setting of immune-mediated disorders. For example, some authors have suggested that CMV, EBV and HSV-1 infections might have a protective influence in the pathogenesis of Celiac disease (CD) (15, 16), while others have demonstrated that the infection with Coxsackie virus B during very early childhood might prevent the development of T1D (17). Among the proposed mechanisms behind their protective role some authors have mentioned the ability of viral agents to shift the immune response from Th1 to Th2 or to cause apoptosis of the immune cells, thus limiting the immune system response and the progression of the autoimmune diseases (18, 19). In addition to a potential protective role in the pathogenesis of immune-mediated disorders, viral infections could also be implied in the clinical improvement or resolution of such conditions. In fact, persistent remission of RA after varicella-zoster virus (VZV) has been described in the past years (20), while in more recent times Barman et al. reported the case of a patient suffering from long-course RA who referred marked improvement of his articular symptoms after the occurrence and resolution of SARS-CoV-2 pneumonia (21). Interestingly, since then and after a 1-year follow-up the patient was considered in persistent and complete remission, with no need of any immunosuppressive therapy. Prior to this case report, remission or significant improvement of chronic diseases following Covid-19 have been reported for oncohematological diseases including mycosis fungoides, follicular and Hodgkin lymphoma (22–24), thus suggesting a potential beneficial influence of the virus even in the inflammatory response involved in cancer biology. Ohadi et al. suggested a possible “oncolytic effect” of SARS-CoV-2 infection to be linked to the typical T-cell depletion induced by the virus itself (24), while Sollini et al. have speculated that in these cases SARS-CoV-2 infection might act as an immunotherapeutic agent by first causing a “flare phenomenon” and later an “abscopal effect”, eventually leading to complete resolution of the tumoral mass (23).

When specifically considering LPP, large part of its pathogenetic mechanisms remains unknown. Nevertheless, it has been suggested that T-cell mediated lichenoid inflammation might be responsible for the development of autoantibodies against type XVII collagen (COL17, BPAG2) leading to the disruption of the hemidesmosomal structure and ultimately to the formation of bullae with both complement-dependent and complement-independent mechanisms (25). The exact nature of the T-cell response in LPP is still unclear, however some authors have suggested that on top of an IFNɣ-mediated Th1 response typical of LP, a dysregulated Th2 response might be necessary for the occurrence of blisters, as seen in patients affected by Bullous Pemphigoid (BP) (2). Similarly to other chronic inflammatory diseases, LP and LPP are likely to share a defect in the physiological process needed for the resolution of inflammation and the molecular pathways involved in the occasional spontaneous resolution of their mucocutaneous lesions are still poorly characterized (26).

Between the development of acute respiratory infection and the resolution of her long-lasting and therapy-resistant mucocutaneous lesions, we cannot exclude the possibility of an underlying causative nexus between the two events. This hypothesis can rely on growing evidence that viral agents, including SARS-CoV-2, can protect from the new onset of immune-mediated diseases and also accelerate their resolution, probably by enhancing innate and/or adaptive immune pathways that are still poorly understood.

By keeping this assumption in mind, we can speculate about the possible immune-mediated mechanisms that could link the two events, based on what clinical and molecular research has so far highlighted concerning the interaction between SARS-CoV-2 and our immune system. We hypothesize that by shifting type Th1 to type Th2 immune response SARS-CoV-2 infection could have first triggered the evolution of LP to LPP in our patient. Then, by other mechanisms already described in the setting of Covid-19 infection, such as consumption of the T cell subpopulations, activation of Treg cells, alteration of IFN I signalling and the production of autoantibodies against immunity-related antigens like IFN-α, the host response to the virus might have interfered with the autoimmune pathogenetic pathways underlying LPP, thus favouring its clinical resolution (8, 27, 28). Moreover, it is worth highlighting that our patient’s immune response to SARS-CoV-2 might have been greatly influenced by previous contacts with the viral agent and the completion of a 3-doses vaccination cycle. It is possible that such factors might have played a booster effect, thus contributing to a rapid clearance of the viral load as well as to the clinical resolution of both respiratory symptoms and mucocutaneous lesions.

Because very little is known concerning the pathogenesis and the involved mechanisms of LPP resolution, we cannot exclude that our patient lesions resolved by chance or because of other unknown factors. In fact, we are aware that from this occasional observation we cannot extrapolate any firm conclusion concerning the pathophysiological mechanisms linking SARS-CoV-2 infection to the resolution of a cutaneous autoimmune disease.

Besides being a single case report, other limitations to our report include that most of the information concerning the disruption of the immune system caused by SARS-CoV-2 infection has been so far collected from hospitalized patients, in which the degree of immunological alterations has often been described as proportional to the severity of the respiratory or systemic involvement (29). Much more is needed to better understand the immunological repercussions of SARS-CoV-2 infection in non-hospitalized and non-severe cases as well as the role of vaccines in the resolution of autoimmune or neoplastic diseases after viral infections.

Nevertheless, we believe that the strikingly close temporal correlation between SARS-CoV-2 infection and the unexpected evolution of the mucocutaneous involvement described in our patient cannot be ignored and that a causative nexus between the two events cannot be dismissed. Our suspicion is strengthened by a growing number of case reports where various conditions of neoplastic and autoimmune nature showed clinical persistent resolution following SARS-CoV-2 infection.

To conclude, we report the probable first case of remission of an immune-mediated, non-neoplastic, cutaneous condition following Covid-19 infection, thus adding a new piece of evidence of an existing correlation between the host’s immune response triggered by the viral agent and the resolution of immune-mediated diseases. Because this hypothesis only relies on isolated cases, large-scale studies are needed to provide more conclusive evidence and a deeper understanding of the underlying immunological mechanisms, including the possible role played in this setting by vaccines.

## Data availability statement

The original contributions presented in the study are included in the article/supplementary material. Further inquiries can be directed to the corresponding author.

## Ethics statement

Written informed consent was obtained from the individual(s) for the publication of any potentially identifiable images or data included in this article.

## Author contributions

MC, AV, LQ, AC, EM, CA, and VRdC were directly involved in patient management. MC, DM, AV, and VRdC produced and drafted the manuscript and revisioned the literature. EDB and BB provided immunoserological analysis. DM and VM were involved in the histopathological analysis and the collection of histopathological pictures. All authors contributed to the article and approved the submitted version.
